# Knowledge-Practice Gaps in Guideline-Based Asthma Management Among Community Pharmacists in Sudan: A Cross-Sectional Study

**DOI:** 10.7759/cureus.115143

**Published:** 2026-08-25

**Authors:** Riham M Hamadouk, Alaa Hamadelnil Ahmed, AlBashir Altaher, Manahil Mohmmed Abdo, Hiba Abdelmunim Suliman Elsheikh, Mohamed Thabit Ahmed, Bashir A Yousef

**Affiliations:** 1 Department of Clinical Pharmacy and Pharmacy Practice, Faculty of Pharmacy, AlMughtaribeen University, Khartoum, SDN; 2 Department of Pharmacy Practice, Faculty of Pharmacy, University of Khartoum, Khartoum, SDN; 3 Department of Clinical Pharmacy, Faculty of Pharmacy, National University, Khartoum, SDN; 4 Department of Pharmacology, Faculty of Pharmacy, Omdurman Islamic University, Khartoum, SDN; 5 Department of Pharmacology, Faculty of Pharmacy, Ege University, Izmir, TUR; 6 Department of Clinical Pharmacy and Pharmacology, Ibn Sina National College for Medical Studies, Jeddah, SAU; 7 Department of Pharmacology, Faculty of Pharmacy, University of Khartoum, Khartoum, SDN

**Keywords:** asthma, asthma management, community pharmacists, gina guidelines, knowledge, practice, sudan

## Abstract

Background: Asthma is a chronic, complex, and multifactorial respiratory disease affecting millions of people worldwide. Despite the availability of effective pharmacological therapies and evidence-based guidelines, asthma control remains suboptimal in many settings. Community pharmacists are well positioned to support asthma management through patient education, inhaler-technique assessment, adherence counselling, and referral when needed, roles increasingly emphasized in contemporary asthma guidelines.

Objectives: This study aimed to assess community pharmacists’ knowledge and practice regarding asthma management in Sudan, identify barriers to optimal care, and evaluate alignment with current asthma guideline recommendations.

Methodology: This observational cross-sectional study was conducted among community pharmacists in Sudan between November 2025 and February 2026. Data were collected using an online structured questionnaire that assessed pharmacists’ knowledge, practice, and perceived barriers related to asthma management. Descriptive statistics were used to summarize participants’ characteristics and response patterns. Inferential analyses were performed using the Pearson Chi-square test to examine associations between knowledge and practice scores and selected pharmacists’ characteristics. A *P*-value <0.05 was considered statistically significant.

Results: A total of 384 community pharmacists participated in the study. More than half were female (*n *= 210, 54.7%), and most held a Bachelor of Pharmacy degree (*n *= 308, 80.2%). The mean knowledge score was 6.63 ± 2.10 out of 9, while the mean practice score was 9.06 ± 3.26 out of 14. Knowledge score was significantly associated with years of professional experience (*χ*^2 ^= 12.851, *P* = 0.045). Practice score was significantly associated with pharmacists’ qualifications (*χ*^2^ = 9.671, *P* = 0.046) and years of experience (*χ*^2^ = 14.204, *P* = 0.027).

Conclusions: Community pharmacists demonstrated generally adequate knowledge of asthma management; however, their practice was only moderate, indicating a significant knowledge-practice gap in implementing current Global Initiative for Asthma (GINA) guideline recommendations. Targeted continuing professional development, practical guideline-based training, and structured asthma-care protocols are needed to strengthen pharmacists’ contribution to asthma management and potentially improve the quality of patient care, adherence to treatment, and asthma control in Sudan.

## Introduction

Asthma is a complex, heterogeneous, and multifactorial chronic respiratory disease characterized by airway inflammation, bronchial hyper-responsiveness, and variable airflow limitation that is often reversible either spontaneously or with treatment [1]. It remains one of the most common chronic non-communicable diseases worldwide, affecting approximately 262 million people and causing an estimated 455,000 deaths in 2019 [2]. Clinically, asthma presents with recurrent respiratory symptoms, including wheezing, shortness of breath, chest tightness, and cough, which vary over time and in intensity [3]. Despite the availability of effective pharmacological therapies and evidence-based international guidelines, asthma remains poorly controlled in many settings, contributing to preventable exacerbations, emergency visits, impaired quality of life, and avoidable healthcare costs [4,5].

The clinical management of asthma is challenging because the disease varies considerably in severity, triggers, inflammatory patterns, treatment response, and patient-related factors [6]. This heterogeneity requires individualized management that considers symptom control, exacerbation risk, inhaler technique, adherence, comorbidities, patient preferences, and access to medicines [6]. The Global Initiative for Asthma (GINA) was established to improve asthma awareness, prevention, diagnosis, and management among healthcare professionals, public health authorities, and patients [7]. GINA provides a comprehensive, adaptable framework for asthma care that can be implemented according to local, regional, and national healthcare contexts [8]. Importantly, recent GINA recommendations no longer support short-acting beta2-agonist (SABA)-only treatment because this approach is associated with increased risk of severe exacerbations and asthma-related mortality. Instead, GINA recommends that adults and adolescents with asthma receive inhaled corticosteroid (ICS)-containing therapy, either as needed in mild asthma or as regular controller therapy in patients with more persistent disease, with treatment adjusted according to control and future risk [9].

Asthma control depends not only on prescribing appropriate medicines but also on sustained adherence, correct inhaler technique, patient education, trigger avoidance, and regular follow-up [10]. International guidelines have emphasized self-management strategies for more than two decades, including written asthma action plans, patient education, and professional guidance from trained healthcare providers [11]. However, many patients continue to use inhalers incorrectly, over-rely on reliever therapy, discontinue controller medicines, or fail to recognize poor asthma control. These gaps are particularly important in primary healthcare and community pharmacy settings, where patients frequently seek advice, refill prescriptions, and discuss medication-related concerns.

Community pharmacists are highly accessible healthcare professionals and are well positioned to contribute to asthma management through medication counselling, inhaler-technique assessment, adherence support, identification of uncontrolled asthma, smoking-cessation advice, and referral when needed [12,13]. During prescription dispensing and medication review services, pharmacists can explain the role of controller and reliever therapies, demonstrate correct inhaler use, address concerns about corticosteroids and adverse effects, detect inappropriate SABA overuse, and encourage adherence to guideline-based treatment [3,13]. Several studies have shown that pharmacist-led interventions can improve asthma symptoms, reduce disease severity, enhance quality of life, and decrease healthcare utilization [14,15].

Despite this important role, the effectiveness of community pharmacists in asthma care depends on their knowledge, practical skills, confidence, and alignment with current guideline recommendations. Evidence from several settings suggests that pharmacists may have variable knowledge of asthma guidelines, inhaler technique, controller therapy, and patient self-management support [14,15]. In Sudan, limited evidence is available regarding community pharmacists’ readiness to deliver guideline-based asthma care, particularly in relation to current GINA recommendations. The primary objective of this study was to assess community pharmacists’ knowledge and self-reported practice regarding asthma management in Sudan, with particular reference to key components of current GINA recommendations, including assessment of asthma severity and symptom control, avoidance of SABA-only therapy, appropriate use of ICS-containing treatment, inhaler-technique assessment, adherence counselling, written asthma action plans, treatment-related adverse effects, symptom monitoring, and follow-up. The secondary objectives were to identify pharmacists’ perceived barriers to providing guideline-based asthma care and to examine the associations of selected demographic and professional characteristics with their knowledge and self-reported practice scores.

## Materials and methods

Study design and setting

This observational cross-sectional study was conducted among community pharmacists in Sudan between November 2025 and February 2026. Data were collected using an online structured questionnaire that assessed community pharmacists’ knowledge and practice regarding asthma management, as well as perceived barriers to providing guideline-based asthma care, with emphasis on alignment with recent GINA recommendations.

Sample size and sampling procedure

A non-probability convenience sampling technique was used. Because of the ongoing armed conflict in Sudan that began in April 2023, updated national data on the number of registered pharmacists in 2025 were unavailable. Therefore, the sample size was calculated using the single-proportion formula:

$n = \frac{Z^2 p(1-p)}{e^2}$

where *n* represents the required sample size, *Z* is the standard normal value corresponding to a 95% confidence level, *p* is the estimated proportion of the target population with the attribute of interest, and *e* is the margin of error. Assuming a 50% response distribution, 95% confidence level, and 5% margin of error, the minimum required sample size was 384 pharmacists.

Data collection tool and procedure

Data were collected using an online self-administered questionnaire developed after an extensive review of the relevant literature and adapted from a previously published study [16]. The original questionnaire was reviewed item by item before use in the present study. Items relevant to community pharmacists’ knowledge and practice of asthma management were retained, while wording and content were modified where necessary to improve clarity and applicability to the Sudanese community pharmacy context. The adapted instrument was also reviewed against the GINA Strategy Report to ensure that the assessed knowledge and practice domains reflected contemporary recommendations relevant to community pharmacists. The questionnaire addresses certain areas of the guideline, including asthma severity and control assessment, the use of SABA-only therapy, ICS use, inhaler technique assessment, asthma action plans, treatment-related side effects, symptom monitoring, and follow-up.

The questionnaire was administered in English. No translation or back-translation procedure was undertaken. The questionnaire consisted of 35 items divided into four sections. The first section included four questions on participants’ sociodemographic and professional characteristics. The second section included nine dichotomous questions assessing pharmacists’ knowledge of asthma management. The third section included fourteen dichotomous questions evaluating pharmacists’ asthma-management practices. The fourth section included a multiple-response question assessing perceived barriers encountered by pharmacists during asthma care.

Validity and reliability assessment

The adapted questionnaire underwent face and content validation before administration. Three pharmacy practice experts independently reviewed the questionnaire for relevance, clarity, comprehensiveness, wording, and suitability for assessing community pharmacists’ knowledge and self-reported practice of asthma management in the Sudanese context. The reviewers also considered the correspondence of the questionnaire items with the selected GINA recommendations assessed in the study. Minor modifications were made to wording and formatting based on their feedback.

A pilot test was then conducted among 20 community pharmacists to evaluate the clarity, feasibility, readability, completion time, and reliability of the questionnaire. Participants in the pilot test were excluded from the final analysis. Based on participants’ feedback, minor wording and formatting adjustments were made to improve the clarity of selected items. Internal consistency was assessed using Cronbach’s alpha. The questionnaire demonstrated acceptable reliability, with Cronbach’s alpha coefficients of 0.79 for the knowledge domain and 0.74 for the practice domain during pilot testing. In the final study sample, internal consistency was reassessed, and Cronbach’s alpha coefficients were 0.708 for the knowledge domain and 0.791 for the practice domain.

Data management and statistical analysis

Responses were exported into Microsoft Excel, checked for completeness and consistency, and then imported into IBM SPSS Statistics, version 26.0 (IBM Corp., Armonk, NY) for analysis. Item-level completeness was assessed for each questionnaire before statistical analysis. All 384 questionnaires included in the final dataset contained complete responses to all applicable questionnaire items, and no missing values were identified. Consequently, no data imputation, pairwise deletion, or other statistical method for handling missing data was required.

Descriptive statistics were used to summarize participants’ demographic and professional characteristics, knowledge items, practice items, and reported barriers. Categorical variables were presented as frequencies and percentages, while knowledge and practice scores were summarized using means and standard deviations.

The knowledge domain included nine items; correct responses were scored as 1 and incorrect or *do not know *responses as 0, giving a total score range of 0-9. Knowledge scores were categorized as poor (0-4), moderate (5-6), or good (7-9). The practice domain included 14 items; appropriate guideline-based practices were scored as 1 and inappropriate or non-performed practices as 0, giving a total score range of 0-14. Practice scores were categorized as poor (0-6), moderate (7-10), or good (11-14). Knowledge and practice scores were analyzed as categorical variables, using frequencies and percentages. Associations between categorical variables were assessed using the Pearson Chi-square test. Cramer's V was calculated to determine the strength of significant associations between the categorical variables, with statistical significance set at *P *< 0.05.

## Results

Demographic characteristics of community pharmacists

A total of 384 community pharmacists completed the questionnaire and were included in the final analysis. All participants provided complete responses to all applicable questionnaire items; therefore, there were no missing or incomplete item-level data, and all 384 participants were included in the analyses without missing-data imputation or exclusion. Females represented 210 (54.7%) of respondents. The most common age group was 26-30 years, accounting for 196 (51.0%) participants. Most pharmacists held a Bachelor of Pharmacy degree (308, 80.2%). Table 1 presents detailed demographic characteristics.

**Table 1:** Sociodemographic characteristics of the community pharmacists (n = 384).

Variable		Frequency (%)
Gender	Male	174 (45.3)
	Female	210 (54.7)
Age groups (years)	21-25	35 (9.1)
	26-30	196 (51.0)
	31-35	95 (24.7)
	36-40	38 (9.9)
	>40	20 (5.2)
Qualification	Bachelor	308 (80.2)
	Master	67 (17.4)
	Ph.D.	9 (2.3)
Years of experience	<2	113 (29.4)
	2-5	81 (21.1)
	6-10	126 (32.8)
	>10	64 (16.7)

Knowledge of community pharmacists regarding asthma management

**Figure 1:**
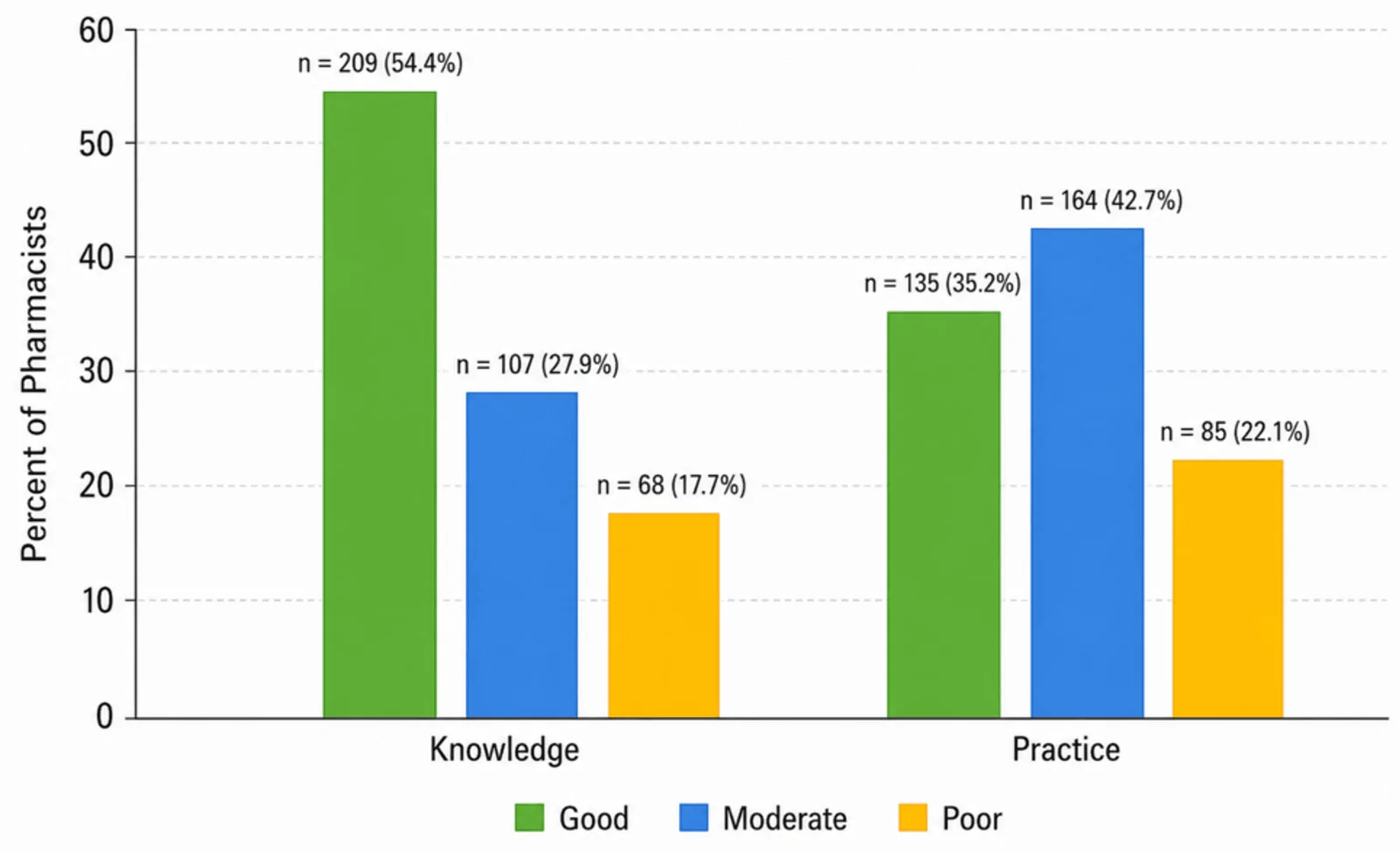
Community pharmacists’ knowledge and practice scores (n = 384).

In the study sample, the knowledge domain demonstrated acceptable internal consistency (Cronbach’s alpha = 0.708). The mean knowledge score was 6.63 ± 2.10, with scores ranging from 1 to 9. Based on the predefined percentage cutoffs, 209 pharmacists (54.4%) demonstrated good knowledge, 107 (27.9%) had moderate knowledge, and 68 (17.7%) had poor knowledge (Figure 1).

Most respondents (365, 95.1%) were aware of the typical respiratory symptoms of asthma, while 19 (4.9%) were not. Most pharmacists (336, 87.5%) reported knowing how to assess asthma severity, compared with 48 (12.5%) who did not. Awareness that ICSs may affect growth in children was reported by 294 respondents (76.6%), while 90 (23.4%) were unaware. The most common knowledge gaps identified by pharmacists were regarding guideline-based pharmacotherapy, as 151 respondents (39.3%) did not know that the GINA no longer recommends SABA monotherapy without ICSs, 140 (36.5%) did not know the recent concerns about SABA use, and 135 (35.2%) did not have knowledge of peak flow meter use. In addition, 132 of the respondents (34.4%) were unaware of recent asthma treatment guidelines. Further details are presented in Table 2.

**Table 2:** Knowledge of the community pharmacists about asthma management (n= 384). ICS, inhaled corticosteroid

Variable	Yes, frequency (%)	No, frequency (%)
Do you know the typical respiratory symptoms of asthma?	365 (95.1)	19 (4.9)
Do you know how to use the peak flow meter?	249 (64.8)	135 (35.2)
Do you know how to assess the severity of your asthma patient?	336 (87.5)	48 (12.5)
Do you know that using steroid inhalation can significantly affect a child's growth?	294 (76.6)	90 (23.4)
Are you aware of the recent asthma treatment guidelines?	252 (65.6)	132 (34.4)
Do you know that the Global Initiative for Asthma (GINA) no longer recommends Short-Acting Beta Agonist (SABA) treatment alone without ICS, even with mild intermittent asthma?	233 (60.7)	151 (39.3)
Do you know the recent concerns about using SABA only?	244 (63.5)	140 (36.5)
Do you know that you should advise patients to avoid using a nebulizer as much as possible for fear of infection transmission during the COVID-19 epidemic?	309 (80.5)	75 (19.5)
Do you know that patients should avoid spirometry with confirmed or suspected COVID-19 cases?	265 (69.0)	119 (31.0)

The Pearson chi-square test showed a statistically significant association between knowledge score and years of professional experience (*χ*² = 12.851, *P *= 0.045). However, the corresponding Cramér’s V indicated a small effect size, suggesting that the magnitude of the association was limited despite statistical significance. Therefore, this finding should be interpreted as an association rather than evidence that professional experience determines pharmacists’ knowledge (Table 3).

**Table 3:** Association between the knowledge of community pharmacists and their demographic characteristics. *Statistically significant at *P *< 0.05. χ², chi-square statistics; df, degrees of freedom; *V*, Cramer’s V

Variables		Poor, frequency (%)	Moderate, frequency (%)	Good, frequency (%)	*χ*²	df	*P*-value	V
Gender	Male (*n* = 174)	35 (20.1)	47 (27.0)	92 (52.9)	1.265	2	0.531	0.057
	Female (*n* = 210)	33 (15.7)	60 (28.6)	117 (55.7)				
Age groups (years)	21-25 (*n* = 35)	6 (17.1)	12 (34.3)	17 (48.6)	3.581	8	0.893	0.068
	26-30 (*n* = 196)	31 (15.8)	55 (28.1)	110 (56.1)				
	31-35 (*n* = 95)	18 (18.9)	28 (29.5)	49 (51.6)				
	36-40 (*n* = 38)	9 (23.7)	8 (21.1)	21 (55.3)				
	>40 (*n* = 20)	4 (20.0)	4 (20.0)	12 (60.0)				
Qualification	Bachelor (*n* = 308)	56 (18.2)	89 (28.9)	163 (52.9)	7.083	4	0.132	0.096
	Master (*n* = 67)	12 (17.9)	13 (19.4)	42 (62.7)				
	Ph.D. (*n* = 9)	0 (0.0)	5 (55.6)	4 (44.4)				
Years of experience	<2 (*n* = 113)	22 (19.5)	32 (28.3)	59 (52.2)	12.851	6	0.045^*^	0.129
	2-5 (*n* = 81)	11 (13.6)	15 (18.5)	55 (67.9)				
	6-10 (*n* = 126)	25 (19.8)	45 (35.7)	56 (44.4)				
	>10 (*n* = 64)	10 (15.7)	15 (23.4)	39 (60.9)				

Practice of community pharmacists toward asthma management

The practice domain demonstrated acceptable internal consistency, with Cronbach’s alpha of 0.791. The mean practice score was 9.06 ± 3.26, with scores ranging from 0 to 14. Practice scores were categorized as poor, moderate, or good. Overall, 164 pharmacists (42.7%) demonstrated moderate practice, 135 (35.2%) had good practice, and 85 (22.1%) had poor practice (Figure 1).

Regarding specific practice items, 261 pharmacists (68.0%) reported taking a detailed history and performing an assessment for patients with asthma, while 292 (76.0%) identified modifiable risk factors associated with poor asthma outcomes. Checking whether patients had a written asthma action plan was reported by 239 pharmacists (62.2%). Most respondents reported checking inhaler technique (323, 84.1%), asking about treatment side effects (313, 81.5%), discussing adherence empathetically with patients (280, 72.9%), and advising regular use of ICSs (287, 74.7%). However, some follow-up and monitoring practices were less frequently reported. Most respondents (270, 70.3%) reported that they did not use peak expiratory flow monitoring for follow-up; 230 pharmacists (59.9%) reported not scheduling follow-up visits; and 215 (56%) reported they did not assess symptom control during the previous four weeks. These findings represent pharmacists’ self-reported practices and were not independently verified through direct observation, dispensing records, or simulated-patient methods. Further details are presented in Table 4.

**Table 4:** Practice of community pharmacists towards asthma management (n = 384).

Variable	Yes, frequency (%)	No, frequency (%)
Do you perform a detailed history examination for asthma?	261 (68)	123 (32)
Do you identify the modifiable risk factors for poor asthma outcomes?	292 (76)	92 (24)
Do you check if the patient has a written asthma plan?	239 (62.2)	145 (37.8)
Do you check the patient's inhalation technique?	323 (84.1)	61 (15.9)
Do you ask patients about their preference in asthma treatment?	277 (72.1)	107 (27.9)
Do you ask the patient about their treatment side effects?	313 (81.5)	71 (18.5)
Do you open an empathic discussion with patients about their adherence?	280 (72.9)	104 (27.1)
Do you advise patients to regularly take their inhaled corticosteroids (ICS), as that might worsen their asthma medications?	287 (74.7)	97 (25.3)
Do you advise patients to discuss with you before stopping any of their medications?	287 (74.7)	97 (25.3)
Do you teach patients about self-monitoring of symptoms?	297 (77.3)	87 (22.7)
Do you assess symptom control over the last four weeks?	169 (44)	215 (56)
Do you use Peak Expiratory Flow Monitoring (PEFM) for the follow-up of asthma patients?	114 (29.7)	270 (70.3)
Do you consider stepping down asthma treatment after proper asthma control?	187 (48.7)	197 (51.3)
Do you schedule a follow-up visit for asthma patients' control every three months	154 (40.1)	230 (59.9)

A statistically significant association was found between practice score and pharmacists’ qualification, *χ*² = 9.671, *P *= 0.046. Practice score was also significantly associated with years of professional experience, *χ*² = 14.204, *P *= 0.027. Nevertheless, Cramér’s V values indicated small effect sizes for both associations, suggesting that the magnitude of these relationships was limited. Accordingly, these results indicate statistical associations only and should not be interpreted as evidence that qualification or professional experience causes better asthma-management practice (Table 5).

**Table 5:** Association between the practice of community pharmacists and their demographic characteristics. *Statistically significant at *P *< 0.05. χ², chi-square statistic; df, degrees of freedom; *V*, Cramer’s V.

Variables		Poor, frequency (%)	Moderate, frequency (%)	Good, frequency (%)	*χ*²	df	*P*-value	*V*
Gender	Male (*n* = 174)	40 (23.0)	80 (46.0)	54 (31.0)	2.438	2	0.296	0.080
	Female (*n* = 210)	45 (21.4)	84 (40.0)	81 (38.6)		8		
Age groups (years)	21-25 (*n* = 35)	5 (14.3)	18 (51.4)	12 (34.3)	5.627	4	0.697	0.085
	26-30 (*n* = 196)	45 (23.0)	82 (41.8)	69 (35.2)				
	31-35 (*n* = 95)	25 (26.3)	36 (37.9)	34 (35.8)				
	36-40 (*n* = 38)	7 (18.4)	20 (52.6)	11 (29.0)				
	>40 (*n* = 20)	3 (15.0)	8 (40.0)	9 (45.0)				
Qualification	Bachelor (*n* = 308)	68 (22.1)	138 (44.8)	102 (33.1)	9.671		0.046^*^	0.112
	Master (*n* = 67)	15 (22.4)	26 (38.8)	26 (38.8)				
	Ph.D. (*n* = 9)	2 (22.2)	0 (0.0)	7 (77.8)				
Years of experience	<2 (*n* = 113)	30 (26.6)	39 (34.5)	44 (38.9)	14.204	6	0.027^*^	0.129
	2-5 (*n* = 81)	17 (21.0)	43 (53.1)	21 (25.9)				
	6-10 (*n* = 126)	31 (24.6)	55 (43.7)	40 (31.7)				
	>10 (*n* = 64)	7 (10.9)	27 (42.2)	30 (46.9)				

Barriers affecting asthma-management practice

The most frequently reported barrier to asthma-management practice was lack of time, reported by 231 pharmacists (60.2%). This was followed by lack of confidence and skills in asthma management, reported by 167 pharmacists (43.5%), and lack of confidence and skills in asthma monitoring, reported by 150 pharmacists (39.1%). The least frequently reported barrier was lack of financial incentive, reported by 74 respondents (19.3%). Additional barriers are summarized in Table 6.

**Table 6:** Barriers to the provision of asthma management according to community pharmacists (n = 384).

Variable	Frequency (%)
Lack of time by the community pharmacist	231 (60.2)
Lack of time by the patient	146 (38.0)
Pharmacists’ perception that it is not their role	109 (28.4)
Patient’s perception that it is not the community pharmacist’s role	125 (32.6)
No financial incentive	74 (19.3)
Lack of pharmacist confidence and skills in asthma management	167 (43.5)
Lack of pharmacist confidence and skills in asthma counseling	140 (36.5)
Lack of pharmacist confidence and skills in asthma monitoring	150 (39.1)

## Discussion

Despite the availability of effective pharmacological therapies, asthma control remains suboptimal in many healthcare settings. Poor adherence, incorrect inhaler technique, inadequate self-management, smoking, poor trigger avoidance, and limited recognition of worsening symptoms all contribute to uncontrolled asthma and preventable exacerbations [17]. Community pharmacists are well positioned to address these gaps because they are accessible healthcare professionals who frequently interact with patients during medication dispensing and counselling [15,18]. This study provides important evidence on Sudanese community pharmacists’ knowledge, practice, and perceived barriers regarding asthma management, with particular attention to alignment with current asthma guideline recommendations.

The findings showed that more than half of pharmacists had good knowledge of asthma management, while most demonstrated moderate practice. Knowledge was particularly strong in relation to asthma symptoms, severity assessment, and avoidance of nebulizer use during COVID-19-related situations. Important knowledge gaps were observed in specialized aspects of guideline-based asthma care. Although GINA guidelines provide a key evidence-based framework for asthma management and help reduce treatment variation, unnecessary hospitalizations, and preventable healthcare costs [19], approximately one-third of pharmacists (132, 34.4%) in this study were unaware of recent asthma treatment guidelines; 36.5% (n=140) of them did not know the recent concerns about SABA use. In addition, 151 (39.3%) of respondents in the present study did not know that SABA monotherapy is no longer recommended without inhaled corticosteroids; 119 (31%) of the pharmacists did not know that patients should avoid spirometry with confirmed or suspected COVID-19 cases, and 135 (35.2%) did not know how to use a peak flow meter. The gap knowledge in our study is better than that reported by Said et al., where nearly two-thirds of pharmacists in the study conducted in Egypt were unaware of updated GINA recommendations, 63.7% of the pharmacists were unaware of GINA recent concerns regarding SABA, and 77.5% of them did not know that GINA no longer recommends SABA alone with ICS, even in cases of mild and intermittent asthma [16]. Previous studies have similarly reported limited use of peak flow meters by pharmacists in asthma monitoring [16,17]. The study findings suggest that community pharmacists possess essential clinical awareness of asthma, consistent with previous evidence from Egypt [16]. Similar findings were reported in Jordan, where healthcare workers, including pharmacists, demonstrated good knowledge of asthma nature, symptoms, and diagnosis [9]. A Nigerian study also reported that most community pharmacists were knowledgeable about the main signs of asthma [20]. However, the present findings contrast with studies from other settings. A Malaysian study reported that community pharmacists failed to recognize several major asthma symptoms [21], while other studies identified poor pharmacist knowledge across multiple domains of asthma management [22]. Similarly, a study from China found that although community pharmacists had positive attitudes toward their professional role, many lacked basic knowledge of pediatric asthma [23]. These differences may reflect variation in pharmacy curricula, continuing professional development, access to guidelines, local pharmacy practice models, and exposure to asthma-care training. The gap in pharmacists’ knowledge about the current GINA guidelines in our study might be due to differences in awareness and access to updated guidelines and suggests ongoing educational asthma programs to give pharmacists up-to-date knowledge.

Regarding practice, pharmacists’ reported asthma-management activities were moderate overall. Many pharmacists reported performing clinically important counselling practices, including checking inhaler technique, asking about side effects, discussing medication adherence, and advising regular inhaled corticosteroid use. These practices indicate a positive contribution to patient education and medication adherence. However, essential follow-up and monitoring activities were less common. The identified gaps in pharmacist practice included the use of peak expiratory flow monitoring for follow-up of patients with asthma (270, 70.3%), scheduling follow-up visits to assess asthma control (230, 59.9%), assessing symptom control over the previous four weeks (215, 56%), considering stepping down asthma treatment after adequate asthma control (197, 51.3%), checking whether patients had a written asthma action plan (145, 37.8%), and taking a detailed history from patients with asthma (123, 32%). These findings were in accordance with a previous study conducted in Egypt, as a low mean weighted score was observed in checking whether patients had a written asthma action plan (2.81), the use of peak expiratory flow monitoring follow-up of asthma patients (3.29), and assessing symptom control over the last four weeks (3.3) [16]. This suggests that pharmacists’ asthma-care activities may remain concentrated around dispensing-related counselling rather than structured long-term disease monitoring.

Peak expiratory flow monitoring can support asthma action plans and help assess asthma control alongside symptoms [24]. Therefore, the low reported use of peak flow monitoring represents an important practice gap. Similar findings have been reported in previous studies, where pharmacists showed limited use of peak flow meters in asthma follow-up [16,17]. Some studies have also reported that peak flow meters were unavailable in many pharmacy workplaces [19,24]. The low use observed in the current study might be related not only to pharmacists’ practice but also to the availability of monitoring devices in the pharmacy, which was not covered in this study.

Although practice in the present study appears better than in some previous studies [16,22], it remains suboptimal and indicates that community pharmacists are not fully utilizing their potential in asthma care. Evidence from systematic reviews and meta-analyses shows that community pharmacy-based asthma self-management support can improve patients’ quality of life, medication adherence, and disease control [25,26]. These findings reinforce the importance of expanding pharmacists’ role beyond dispensing to include structured counselling, inhaler-technique assessment, adherence support, symptom monitoring, and referral of uncontrolled cases. It was reported that practice guidelines, the structure and organization of healthcare, and patient and healthcare professional attitudes regarding asthma self-management vary in developing countries. In addition, there are variations in community pharmacists' willingness and ability to provide services in terms of personnel and time availability, as well as the lack of payment for such services [25].

In Sudan, as one of the developing countries, patients’ access to healthcare is impacted by their financial status [27], and most of the patients rely on community pharmacies for their medical care, which might include asthma patients. Because asthma is both episodic and chronic, pharmacists' interventions in creating patient self-management strategies are crucial for long-term asthma control [16]. Expanding pharmacy services to include asthma management in this setting requires awareness and practical adherence to the GINA guidelines, improving access to peak flow meters, providing hands-on training, integrating monitoring tools into pharmacy-based asthma services, and continuing professional development. Such measures may strengthen the contribution of community pharmacists to asthma care by enhancing pharmacists’ ability to identify uncontrolled asthma and refer patients appropriately.

The Pearson chi-square test showed that years of professional experience were significantly associated with knowledge scores. Pharmacists with two to five years of experience showed better knowledge than other experience groups. This may reflect more recent academic exposure, greater familiarity with updated guidelines, or early-career motivation for continuing education. However, this association should be interpreted cautiously because knowledge may also be influenced by postgraduate training, workplace environment, access to professional development, and prior hospital or clinical experience, which were not fully assessed in this study.

Practice score was not significantly associated with age or gender. However, pharmacists’ qualifications and years of professional experience were significantly associated with practice. Pharmacists with higher academic qualifications, particularly postgraduate degrees, demonstrated better practice levels than those with bachelor’s degrees. Similarly, pharmacists with more professional experience were more likely to demonstrate good practice. These findings may suggest that advanced education and accumulated clinical exposure may be related to pharmacists’ confidence, counselling skills, and ability to deliver pharmaceutical care. Continuing professional development programs, practical guideline-based workshops, and integration of pharmacists into multidisciplinary asthma-care pathways could help improve the quality of asthma services in community pharmacies.

The study also identified several barriers that may limit pharmacists’ ability to provide optimal asthma care. Lack of time was the most common barrier, followed by lack of confidence and skills in asthma management and monitoring. Previous studies have consistently identified insufficient time, limited training, and low confidence as major obstacles to pharmacist-led asthma services [16,28]. Patient-related barriers were also reported, including limited patient time and the perception that asthma care is not part of the pharmacist’s role. Similar barriers have been documented in other settings [28,29]. These findings highlight both professional and system-level challenges that must be addressed before community pharmacy asthma services can be implemented effectively.

Public awareness of pharmacists’ clinical role is also essential. Previous research has shown that patients may have limited understanding of pharmacists’ education, training, and capacity to support chronic disease management [30]. Therefore, interventions should target both pharmacists and the public. Training pharmacists without improving patient acceptance and health-system integration may limit the impact of pharmacy-based asthma services. Public education campaigns, professional advocacy, and formal recognition of community pharmacists as asthma-care providers may improve service uptake and patient trust.

Overall, this study highlights a clinically important knowledge-practice gap among Sudanese community pharmacists. While general asthma knowledge was acceptable, practice was not fully aligned with current guideline-based recommendations, particularly regarding structured follow-up, assessment of recent symptom control, peak flow monitoring, and asthma action plans. Addressing these gaps through training, service redesign, guideline dissemination, and improved pharmacy resources could strengthen pharmacists’ contribution to asthma control in Sudan.

This study has several limitations. First, the cross-sectional design prevents causal inference between pharmacists’ characteristics and their knowledge or practice. Accordingly, statistically significant associations observed in this study should not be interpreted as indicating that professional experience or academic qualification causes differences in knowledge or practice. Moreover, the small effect sizes observed for the significant associations indicate that these relationships were limited in magnitude. Second, the use of convenience sampling and online data collection may have introduced selection bias, as pharmacists with internet access or greater interest in asthma care may have been more likely to participate. Therefore, despite achieving the calculated sample size, the study sample cannot be considered nationally representative, and the results should not be generalized to all community pharmacists in Sudan. Third, the study relied on self-reported responses, which may be affected by recall bias and social desirability bias; therefore, reported practices may not fully reflect actual pharmacy-based behavior, and the extent to which pharmacists’ knowledge is applied in their actual practice could not be directly determined. Fourth, some potentially important variables were not assessed, including prior asthma training, continuing professional development, postgraduate clinical exposure, hospital experience, workload, availability of peak flow meters, and access to GINA guidelines. Fifth, the questionnaire contains items related to COVID-19, which may have reduced its relevance to the current context of asthma management. Finally, although the sample size met the calculated minimum requirement, the findings may not be fully generalizable to all community pharmacists in Sudan, particularly given the ongoing conflict and disruption of pharmacy services.

## Conclusions

This study found that Sudanese community pharmacists generally had good knowledge of asthma management; however, a substantial proportion had moderate or poor knowledge. Their reported practice was moderate and not fully aligned with recent GINA guideline recommendations. Key practice gaps included limited assessment of recent symptom control, low use of peak flow monitoring, incomplete follow-up scheduling, and inconsistent implementation of asthma action plans. Knowledge was significantly associated with years of professional experience, while practice was significantly associated with qualification and years of experience; however, because the study had a cross-sectional design, these associations do not imply causality. The main barriers to optimal asthma care were lack of time, limited confidence, and insufficient skills in asthma management and monitoring.

These findings support the need for structured continuing professional development programs focused on current asthma guidelines, inhaler technique, asthma action plans, adherence counselling, and monitoring of disease control. Future research should evaluate actual pharmacy practice using simulated-patient methods, direct observation, or intervention-based designs to better identify practical gaps and measure the impact of training on patient outcomes.
